# Association between objectively measured physical activity and body mass index with low back pain: a large-scale cross-sectional study of Japanese men

**DOI:** 10.1186/s12889-018-5253-8

**Published:** 2018-03-09

**Authors:** Yuko Hashimoto, Ko Matsudaira, Susumu S. Sawada, Yuko Gando, Ryoko Kawakami, Robert A. Sloan, Chihiro Kinugawa, Takashi Okamoto, Koji Tsukamoto, Motohiko Miyachi, Hisashi Naito

**Affiliations:** 1 Faculty of Health and Sports Science, Graduate School of Juntendo University, 1-1 Hirakagakuendai, Inzai, Chiba, 270-1695 Japan; 2 Department of Medical Research and Management for Musculoskeletal pain, 22nd Century Medical and Research Center, Faculty of Medicine, The University of Tokyo Hospital, 7-3-1, Hongo, Bunkyo-ku, Tokyo, 113-8655 Japan; 3 Department of Physical Activity Research, National Institutes of Biomedical Innovation, Health and Nutrition, 1-23-1 Toyama, Shinjuku-ku, Tokyo, 162-8636 Japan; 4 Faculty of Sport Sciences, Waseda University, 2-579-15 Mikajima, Tokorozawa city, Saitama, 359-1192 Japan; 5 Department of Psychosomatic Internal Medicine, Graduate Medical and Dental School, Kagoshima University, 8-35-1 Sakuragaoka, Kagoshima city, Kagoshima, 890-8544 Japan; 6 Tokyo Gas Co., Ltd., 1-5-20 Kaigan, Minato-ku, Tokyo, 105-8527 Japan

**Keywords:** Accelerometry, Epidemiology, Physical activity, Low back pain, Body mass index

## Abstract

**Background:**

The relationship between the combination of physical activity (PA) and body mass index (BMI) with low back pain (LBP) is unclear. The present study offers a cross-sectional assessment of how combinations of PA and BMI are related to LBP in Japanese men.

**Methods:**

Participants were 4022 Japanese men (mean age = 47) who underwent regular clinical examinations. PA was measured using a uniaxial accelerometer and divided into tertiles (PA_high_, PA_middle_, PA_low_). A self-administered questionnaire was used to report on persistent LBP experience, drinking and smoking habits, and any existing lifestyle diseases. After covariance adjustment, a logistic regression model was used to assess how combinations of PA and BMI are related to persistent LBP.

**Results:**

428 of the participants had persistent LBP. A clear negative dose-response relationship was found between PA levels and persistent LBP (P for linearity = 0.012). Regarding BMI, odd ratios were shown to be higher in the overweight/obese category (BMI ≥ 25 kg/m^2^) than for the normal weight category (BMI < 25 kg/m^2^). When the PA_high_ was taken as the reference in the normal weight category, odds ratios for PA_low_ and PA_middle_ in the normal weight category were shown to be high. Moreover, in the overweight/obese category, odd ratios for every fitness level were also high as for the normal weight category.

**Conclusion:**

The present study showed that both PA and BMI are related to persistent LBP. Also, the prevalence of persistent LBP became higher when PA_low_ and high BMI are combined rather than the group of PA_high_ and low BMI combination.

**Electronic supplementary material:**

The online version of this article (10.1186/s12889-018-5253-8) contains supplementary material, which is available to authorized users.

## Background

Low back pain (LBP) ranks the highest in symptom that affect years lived with disability and currently is a health issue worldwide [1]. In national health and nutrition surveys in Japan as well, LBP is ranked highest for men among those reporting subjective symptoms for disease or injury [2]. LBP affects not only health but also productivity and leads to loss of labor and decline in workforce and productivity, thus causing economic losses to society [3].

The lack of physical activity (PA) [4] and body mass index (BMI) [5] have been reported to be related to a variety of diseases, and many reports exist on the relationship between PA and LBP [6–12] as well as the relationship between BMI and LBP [11–15]. However, study results for both relationships are diverse, and the relationship for each with LBP is not quite clear. The reason behind the variation in study results is perhaps due to the differences in measuring methods for the various indices or differences in the studied populations. Most studies have used self-report questionnaires to measure PA but some have reported the challenges of the subjective method [16, 17]. Also, only few small-scale studies have used objectively measured PA via accelerometer [18–20]. Furthermore, no studies observed Asian populations, and there are no investigations regarding the combinations of PA and BMI related to LBP.

There are some related plausible mechanisms regarding the relationships between PA and obesity with LBP. Both PA and obesity yield some different endogenous substances. From previous studies, PA seems to contribute mood change and pain reduction, [21, 22] and obesity, which yields enlarged fat cell disrupt the balance of the secretion system and are possibly related to pain [23].

Based on the relationships and mechanisms of PA, obesity, and pain above, we set up a hypothesis that there is a relationship between the combination of low PA and high BMI with high prevalence of LBP. Therefore, we examined how combinations of PA and BMI are related with LBP in over 4000 Japanese men using an accelerometer to objectively measure the amount of physical activity.

## Methods

### Participants

**Fig. 1:**
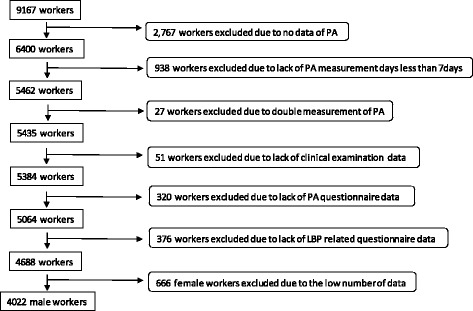
Flowchart of the sample selection for the present study

Participants were 9167 workers of a single company in metropolitan Tokyo and the ratio of blue-collar to white-collar workers was approximately 6:4. The participants underwent a clinical examination under the Industrial Safety and Health Act in Japan between September 2009 and August 2010. Out of these 9167 participants, the focus was on 6400 participants who measured PA with an accelerometer. Then, 938 who measured PA using the accelerometer for less than seven days were excluded. 27 participants were excluded because of double measurement of physical activity, then 51 participants were excluded due to lack of clinical examination data. Participants who did not fully complete the questionnaire relating to their PA measurement (*n* = 320) and LBP (*n* = 376) were excluded. Moreover, since the number of female’s data was low (*n* = 666), we excluded them from the analysis. Finally, the examined population for analysis in this research consisted of 4022 men (Figure 1).

In our observational study, the participants gave written informed consent for research use and publication of his/her data before commencement of the study and the clinical examinations were done under the Industrial Safety and Health Act and related laws in Japan. This study was approved by the ethics committee of the National Institutes of Health and Nutrition (290–01).

### Clinical examination

At the clinical examination, height (m) and body weight (kg) were measured in light clothes without shoes, and all participant’s height and weight were recorded by the same scale with the same condition. BMI was obtained from height and weight measurements (weight divided by height squared) by questionnaire, and are divided into two categories using the WHO BMI standard, that is normal weight (< 25 kg/m2) and overweight/obese (≥ 25 kg/m2). Participants were asked about drinking and smoking habits, and if they were receiving treatment at the time for hypertension, dyslipidemia, or diabetes.

### Measurement of physical activity

PA was measured using a Lifecorder PLUS (Suzuken Co., Ltd), a uniaxial accelerometer, and it determines the number of steps and the physical activity level (1 to 9) per day. Suitability and reliability of the accelerometer used in the present study have been confirmed in previous studies [24, 25]. Accelerometers were distributed to participants due for a clinical examination two months prior. Also, from the participants who agreed to participate in the study, median values for each participant were collected concerning moderate-vigorous PA (≥ 3 METs) times (min/day) as indicators of PA [26]. In this study, based on Kumahara’s criteria [24], the intensities were defined as 1 to 3, 4 to 6, and 7 to 9 as light (< 3 METS), moderate (3-6METS), and vigorous (> 6 METS) intensity of physical activity and PA was considered as ≥3 METs, which is more than moderate-vigorous PA. We did not consider bout length. Participants who consented to take part in the study were asked to wear the accelerometer for two weeks or more and 12 h or more per day [27]. The criteria for data to be selected for the use were set at a minimum of seven days where the acceleration was detected for 10 h or more per day [28].

### Ascertainment of low back pain

Experience of LBP was examined using a self-administered questionnaire at the time of the clinical examination. Under the question concerning objective symptoms, the following three responses were provided as options for LBP: None, Sometimes (Intermittent), and All the time (Persistent). Based on previous studies, persistent LBP was considered as continuous pain [29, 30]. Therefore, we defined participants who responded “All the time” on the self-administered questionnaire as people who have persistent LBP, and excluded people who answered sometimes (Intermittent).

### Statistical analysis

First, we compared characteristics for participants with persistent LBP and those without. PA for all participants were divided into three categories (PA_high_, PA_middle_, PA_low_) based on tertiles. Each classification was≦49.1 min/day (high), 33.9–49.1 minuets/day (middle), and≧33.9 min/day (low) respectively. By combining two BMI categories (Normal weight, Overweight/obese) and three PA level categories, a total of six categories were established (Normal weight - PA_high_, Normal weight - PA_middle_, Normal weight - PA_low_, Overweight/obese - PA_high_, Overweight/obese - PA_middle_, Overweight/obese - PA_low_), and the characteristics of these six categories were compared. Next, to study the relationship between persistent LBP with PA and BMI, respectively, logistic regression analysis was performed with the presence of persistent LBP as the dependent variable and PA (three categories) or BMI (two categories) as the independent variables. The odds ratios (ORs) and the 95% confidence intervals (CIs) adjusted for age were calculated. Moreover, the ORs adjusted for hypertension (yes, no), dyslipidemia (yes, no), diabetes (yes, no), drinking habits (drinker, nondrinker), and smoking habits (nonsmoker, smoker, former smoker) were obtained. As a final adjustment, ORs were obtained for PA with added BMI (kg/m^2^) and BMI with added PA (minutes/day). To assess the relationship between the six categories combining PA and BMI and persistent LBP, logistic regression analysis was performed with persistent LBP as a dependent variable and the six categories with combinations of PA and BMI as independent variables. Then 95% CIs and ORs adjusted for confounding factors were calculated. Additionally, to confirm whether or not there was any effect modification of PA or BMI, the existence of interaction was confirmed by inserting product terms PA (minutes/day) and BMI (kg/m^2^) into the model. All statistical analyses were performed using SPSS Statistics version 21(SPSS Japan Inc., Tokyo, Japan), and a two-tailed *P* value less than .05 was considered to be statistically significant.

## Results

Out of the 4022 participants, 428 had persistent LBP. Participant characteristics for those with and without persistent LBP are shown in Table 1. Age and BMI in participants with persistent LBP were high, whereas PA was low. Smoking and drinking rates and overall ratios of lifestyle disease tended to be high in participants with persistent LBP.

**Table 1:** Characteristics of Japanese Men According to Persistent LBP

Characteristics	Total	With Persistent LBP	Without Persistent LBP
N	4022	428	3594
Age, years	47 (10)	49 (9)	47 (10)
BMI, kg/m^2^	23.8 (3.2)	24.2 (3.5)	23.8 (3.1)
Physical activity, minutes/day	42.7 (18.5)	40.5 (17.6)	43.0 (18.5)
Drinking, %			
Nondrinker	14.5	14.0	14.6
Drinker	85.5	86.0	85.4
Smoking, %			
Nonsmoker	35.9	30.8	36.5
Smoker	36.6	38.1	36.4
Former smoker	27.5	31.1	27.0
Lifestyle-related diseases, %			
Hypertension	17.4	20.3	17.0
Dyslipidemia	8.9	9.8	8.8
Diabetes	5.4	7.7	5.1

Data are means (SD) or %

*LBP* Low back pain, *BMI* Body mass index, *SD* Standard deviation

Participant characteristics for the six categories with PA/BMI combinations are shown in Table 2. This shows that in the two obesity status categories, the number of people in the normal weight category is twice the number in the overweight/obese category. Moreover, age tends to be higher in the overweight/obese category than in the normal weight category across the PA_low_, PA_middle_, and PA_high_. The absolute risk of persistent LBP was the highest when overweight/obese and PA_low_ are combined. Regardless of normal weight or overweight/obese, the absolute risk of persistent LBP increased with the lowering of physical activity levels. No constant trend was found for drinking rates, but smoking rates were highest in the category with PA_low_, both in the overweight/obese and normal weight categories. High values were shown for hypertension, dyslipidemia, and diabetes in the overweight/obese category.

**Table 2:** Characteristics of participants according to PA levels and BMI

Obesity status	Normal weight (BMI < 25)			Overweight/Obese (BMI ≥ 25)		
Physical activity levels	High	Middle	Low	High	Middle	Low
N	935	927	906	402	416	436
Persistent low back pain (%)	7.9	11.4	10.3	10.2	13.0	13.8
Age, years	47 (10)	46 (10)	47 (10)	49 (9)	47 (9)	49 (9)
BMI, kg/m^2^	22.1 (1.8)	22.3 (1.7)	22.2 (1.8)	27.4 (2.3)	27.2 (2.2)	27.7 (3.0)
Physical activity, minutes/day	62.9 (14.0)	41.4 (4.3)	24.2 (7.3)	62.6 (13.3)	41.5 (4.5)	23.4 (7.4)
Drinkers (%)	85.5	84.6	86.1	86.3	88.2	82.8
Smokers (%)	32.7	34.8	39.4	33.6	39.2	43.1
Hypertension (%)	12.9	9.0	15.8	26.6	30.3	27.1
Dyslipidemia (%)	6.6	5.0	7.5	14.2	15.4	14.2
Diabetes (%)	3.5	3.9	3.0	11.2	8.2	9.6

Data are means (SD) or %

*BMI* Body mass index, *SD* Standard deviation

The ORs for persistent LBP by PA and by obesity status are shown in Table 3. PA_low_ showed higher ORs than PA_high_, and a clear negative dose-response relationship was found between PA and having persistent LBP (P for linearity = 0.012). For BMI, the high category (overweight/obese; BMI ≥ 25 kg/m^2^) showed higher ORs than the low category (normal weight; BMI < 25 kg/m^2^). No significant interaction was observed between PA and obesity status (P for interaction = 0.477).

**Table 3:** Multivariable-adjusted odds ratio for persistent LBP by PA levels and BMI

	N	With Persistent LBP	Persistent LBP per 100 men	Age-adjusted OR (95% CI)	Multivariable^a^ OR (95% CI)	Multivariable^b^ OR (95% CI)
Physical activity levels						
High	1337	115	8.6	1.00 (reference)	1.00 (reference)	1.00 (reference)
Middle	1343	160	11.9	1.46 (1.14–1.89)	1.46 (1.13–1.88)	1.46 (1.13–1.88)
Low	1342	153	11.4	1.37 (1.06–1.76)	1.36 (1.05–1.76)	1.35 (1.04–1.74)
P for linearity				0.009	0.009	0.012
Obesity status						
BMI < 25	2768	273	9.9	1.00 (reference)	1.00 (reference)	1.00 (reference)
BMI ≥ 25	1254	155	12.4	1.26 (1.02–1.55)	1.22 (0.99–1.52)	1.22 (0.98–1.51)

*LBP* Low back pain, *BMI* Body mass index, *OR* Odds ratio, *CI* Confidence interval

^a^Adjusted for age (years), hypertension (yes, no), dyslipidemia (yes, no), diabetes (yes, no), drinking (nondrinker, drinker), and smoking (nonsmoker, smoker, former smoker)

^b^Further adjusted for body mass index (kg/m^2^) for physical activity categories or physical activity (minutes/day) for obesity status

The ORs for persistent LBP in the six categories with PA and obesity status combinations are shown in Table 4. When taking PA_high_ at normal weight (BMI < 25 kg/m^2^) as the standard, PA_middle_ and PA_low_ ORs showed to be high for normal weight. Furthermore, similarly high ORs as for normal weight are shown for PA_high_, PA_middle_, and PA_low_ for overweight/obese (BMI ≥ 25 kg/m^2^), and the PA_low_ OR was the highest at 1.75 (95% CI, 1.22–2.53).

**Table 4:** Multivariable-adjusted odds ratio for persistent LBP according to combined PA levels and BMI at baseline

Obesity status	Physical activity levels	N	With Persistent LBP	Persistent LBP per 100 men	Age-adjusted OR (95% CI)	Multivariable^a^ OR (95% CI)
Normal weight (BMI < 25)	High	935	74	7.9	1.00 (reference)	1.00 (reference)
	Middle	927	106	11.4	1.53 (1.12–2.09)	1.52 (1.11–2.08)
	Low	906	93	10.3	1.33 (0.97–1.83)	1.33 (0.96–1.83)
Overweight/obese (BMI ≥ 25)	High	402	41	10.2	1.28 (0.86–1.92)	1.25 (0.83–1.87)
	Middle	416	54	13.0	1.72 (1.19–2.50)	1.68 (1.15–2.44)
	Low	436	60	13.8	1.80 (1.26–2.59)	1.75 (1.22–2.53)

*LBP* Low back pain, *BMI* Body mass index, *OR* Odds ratio, *CI* Confidence interval

^a^Adjusted for age (years), hypertension (yes, no), dyslipidemia (yes, no), diabetes (yes, no), drinking (nondrinker, drinker), and smoking (nonsmoker, smoker, former smoker)

Additionally, the results of the combined “None/Sometime (intermittently)” and “All the time (Persistent)” group of LBP are provided in an additional file as a sensitivity analysis (Additional file 1).

## Discussion

This study shows a cross-sectional assessment of the relationship between objectively measured PA and BMI, with persistent LBP in 4022 Japanese men. A negative dose-response relationship was found between PA and persistent LBP, and a positive relationship was found between BMI and persistent LBP. Regardless of normal weight or overweight/obese, the absolute risk of persistent LBP slightly increased with the lowering of physical activity level (from 7.9% to 13.8%) and no support was obtained for effect modification.

In research that reports similar results to this study regarding the relationship between PA and LBP, Ryan CG et al. report that patients with chronic LBP perform fewer steps per day than patients with non-chronic LBP and also have shorter walking periods [18]. Lin et al. also report in a systematic review that patients with chronic LBP with a high level of disability tend to have low PA [6]. Moreover, some studies report that participants that have moderate levels of PA have few instances of chronic LBP patients [8, 9]. This study demonstrates similar results to these studies. On the other hand, Kamada et al. [10] did not find a clear relationship between PA and LBP. It is believed that since this study evaluated PA using a questionnaire, PA was not ascertained accurately. This study uses actual accelerometer measurements of PA, and we believe that the relationship between PA and LBP is assessed accurately.

With regard to the relationship between BMI and LBP, the HUNT study [14] reports, as the present study, a significant positive relationship between BMI and persistent LBP. In the HUNT study, which looks at Western populations, BMI distribution differs greatly from the male participants in our study. When male participants are divided into two categories using the WHO BMI standard, that is normal weight (< 25 kg/m^2^) and overweight/obese (≥ 25 kg/m^2^), the proportion for participants of the HUNT study were 34% and 71%, respectively, whereas in this study, they significantly differ at 69% and 31%. However, since a positive relationship between BMI and LBP is observed for both of them, regardless of the difference with Western countries and Asia, it is believed that a relative tendency to obesity, rather than absolute BMI values, is a risk factor for having persistent LBP.

First, the following are two plausible mechanisms to explain the study’s result of the relationship between PA and LBP: 1) PA is low because people are suffering from persistent LBP, and 2) people have persistent LBP caused by the fact that PA is low. Concerning the first, one factor that can be offered is fear-avoidance beliefs (FAB), when patients excessively avoid activities due to anxiety and fear towards pain. Wertli et al. says that the reduction of FAB in patients may avoid delayed recovery and chronicity [31]. For the reason that patients experience persistent LBP because of low PA, the second point above, it can be considered to be pain relief due to PA. Relevant endogenous substances include endogenous cannabinoids and opioids, which are reported to contribute to post-exercise mood changes and affect the central nervous system mechanisms of pain modulation [21, 22]. Reports also exist that light to moderate spare-time PA is effective in preventing LBP [32]. Furthermore, another report states that PA on its own or in combination with education is effective in preventing LBP [33].

Next, the following two are plausible mechanisms to explain the study’s result of the relationship between obesity and persistent LBP: 1) a biomechanics perspective and 2) a relationship with an endogenous substance. The first is the biomechanics perspective of stress on the spine (intervertebral discs) caused by enlarged stomach due to obesity, as seen in excessive thoracic kyphosis or lumbar lordosis caused by increased downward center of gravity in obese people [34]. Flexing the upper body forward further increases the gravitational force and increases stress on the intervertebral discs, which forms the main reason for the increased power that is required from the back muscle groups. Regarding the relationship with endogenous substances, there is a possible relationship between pain and proinflammatory cytokines, which are induced by adipokines secreted by enlarged fat cells. Typical of these are tumor necrosis factor-alpha **(**TNF-α) and interleukin-6 (IL-6), and blood levels of IL-6 are thought to be elevated in obese patients [23]. Accordingly, secretion defects for endogenous substances such as adipokines and proinflammatory cytokines in enlarged fat cells disrupt the balance of the secretion system and are possibly related to pain. Additionally, there is myokine, an endogenous substance that promotes the decomposition of fat which is secreted by exercised muscles. Myokines extend to several tens of types, and their action in controlling systemic and slight chronic inflammation has been confirmed [35].

Based on the four mechanisms described above, fat burning and pain relief through PA is believed to be a few of the mechanisms of PA preventing LBP. It is possible that this mechanism explains the relationship of a higher LBP ratio in PA_low_ that was shown in this study. Moreover, there is also the possibility that, regardless of the standard of obesity, maintaining current weight and avoiding further weight gain can prevent persistent LBP. Due to the cross-sectional study design, any causal relationship cannot be mentioned, but it suggests that if people have persistent LBP caused by low PA and being obese, respectively, encouraging people to engage in weight control including physical activity such as walking as part of daily life will not only help prevent lifestyle diseases, metabolic syndrome, cardiovascular diseases, and mental health problems in Japanese men but will also contribute to the prevention of LBP.

The present study has some limitations. First, subjects were employees of a single company and it is based only on data for men from an industry with a limited number of people, so there are limits to its generalizability. Moreover, the question of LBP was very simple and definition of persistent LBP does not consider degrees of chronicity, disability, specific body area, or symptoms, so reliability and validity of these data have not been confirmed. Also, accelerometer was performed for more than 2 weeks, the symptomatology could possibly have disappeared. However, since we did not include participants who answered ‘sometimes (intermittent)’ for the analysis, we consider the participants we analyzed have a high possibility of presenting persistent LBP. On the other hand, our strength is that measurements were taken using an accelerometer, resulting in objective indices for PA, and additionally that the study was on an unprecedented large scale. To determine if PA and obesity status contribute to the prevention of persistent LBP, we hope to conduct a longitudinal study of the relationship between amount of PA, obesity status, and LBP in the future. Furthermore, we hope to conduct a study that includes various populations including women and increases generalizability and provides a more accurate grasp of LBP experience.

## Conclusion

This study shows that both moderate-vigorous PA (≥ 3 METs) and BMI are related to having persistent LBP. Also, the prevalence of persistent LBP became higher when PA_low_ and high BMI are combined rather than the group of PA_high_ and low BMI combination.

## Additional file

Additional file 1 **Table S1-B**: Sensitivity Analysis, **Table S2-B**: Sensitivity Analysis. Sensitivity analysis of the results of the combined “None/Sometime (intermittently)” and “All the time (Persistent)” group of LBP compared to the “All the time (Persistent)” group of LBP. (DOCX 24 kb)

## Abbreviations

BMI: Body mass index
CI: Confidence interval
FAB: Fear-avoidance beliefs
IL-6: Interleukin-6
LBP: Low back pain
OR: Odds ratio
PA: Physical activity
TNF-α: Tumor necrosis factor-alpha
WHO: World Health Organization
