# Supplementary material for: Association between objectively measured physical activity and body mass index with low back pain: a large-scale cross-sectional study of Japanese men
Source: BMC Public Health. 2018 Mar 9;18:341. doi: 10.1186/s12889-018-5253-8 (PMC5845261; doi:10.1186/s12889-018-5253-8)
Supplement: Supplementary file 1 — Table S1-B: Sensitivity Analysis, Table S2-B: Sensitivity Analysis. Sensitivity analysis of the results of the combined “None/Sometime (intermittently)” and “All the time (Persistent)” group of LBP compared to the “All the time (Persistent)” group of LBP. (DOCX 24 kb) [file 12889_2018_5253_MOESM1_ESM.docx]

**Table S1-B: Sensitivity Analysis**

Multivariable-adjusted odds ratio for LBP by PA levels and BMI.

|  | N | With LBP | | LBP per 100 men | Age-adjusted OR (95% CI) | | Multivariable^a^ OR (95% CI) | Multivariable^b^ OR (95% CI) |
| --- | --- | --- | --- | --- | --- | --- | --- | --- |
| Physical activity levels | | | | | | | | |
| High | 1337 | 680 | | 50.9 | 1.00 (reference) | | 1.00 (reference) | 1.00 (reference) |
| Middle | 1343 | 768 | | 57.2 | 1.33 (1.14−1.55) | | 1.32 (1.13−1.54) | 1.32 (1.13−1.54) |
| Low | 1342 | 773 | | 57.6 | 1.32 (1.13−1.53) | | 1.29 (1.11−1.51) | 1.29 (1.10−1.50) |
| P for linearity |  |  | |  | 0.002 | | 0.005 | 0.007 |
|  |  |  | |  |  | |  |  |
| Obesity status | | |  | | |  | | |
| BMI < 25 | 2768 | 1490 | | 53.8 | 1.00 (reference) | | 1.00 (reference) | 1.00 (reference) |
| BMI ≥ 25 | 1254 | 731 | | 58.3 | 1.15 (1.00−1.32) | | 1.12 (0.98−1.29) | 1.12 (0.97−1.29) |

LBP, low back pain; BMI, body mass index, OR, odds ratio; CI, confidence interval.

^a^ Adjusted for age (years), hypertension (yes, no), dyslipidemia (yes, no), diabetes (yes, no), drinking (nondrinker, drinker), and smoking (nonsmoker, smoker, former smoker).

^b^ Further adjusted for body mass index (kg/m^2^) for physical activity categories or physical activity (minutes/day) for obesity status.

**Table S2-B: Sensitivity Analysis**

Multivariable-adjusted odds ratio for LBP according to combined PA levels and BMI at baseline.

| Obesity status | Physical activity levels | N | With LBP | LBP per 100 men | Age-adjusted OR (95% CI) | Multivariable^a^ OR (95% CI) |
| --- | --- | --- | --- | --- | --- | --- |
| Normal weight  (BMI < 25) | High | 935 | 472 | 50.5 | 1.00 (reference) | 1.00 (reference) |
|  | Middle | 927 | 508 | 54.8 | 1.43 (1.13−1.80) | 1.39 (1.10−1.76) |
|  | Low | 906 | 510 | 56.3 | 1.62 (1.28−2.06) | 1.56 (1.23−1.99) |
| Overweight/obese  (BMI ≥ 25) | High | 402 | 208 | 51.7 | 1.00 (0.79−1.27) | 0.99 (0.78-1.25) |
|  | Middle | 416 | 260 | 62.5 | 1.27 (1.05−1.52) | 1.25 (1.04−1.50) |
|  | Low | 436 | 263 | 60.3 | 1.22 (1.01−1.46) | 1.22 (1.02−1.47) |

LBP, low back pain; BMI, body mass index, OR, odds ratio; CI, confidence interval.

^a^ Adjusted for age (years) , hypertension (yes, no), dyslipidemia (yes, no), diabetes (yes, no), drinking (nondrinker, drinker), and smoking (nonsmoker, smoker, former smoker).
